# Investigation of Nano-Bio Interactions within a Pancreatic Tumor Microenvironment for the Advancement of Nanomedicine in Cancer Treatment

**DOI:** 10.3390/curroncol28030183

**Published:** 2021-05-24

**Authors:** Abdulaziz Alhussan, Kyle Bromma, Ece Pinar Demirci Bozdoğan, Andrew Metcalfe, Joanna Karasinska, Wayne Beckham, Abraham S. Alexander, Daniel J. Renouf, David F. Schaeffer, Devika B. Chithrani

**Affiliations:** 1 Physics and Astronomy, University of Victoria, Victoria, BC V8P 5C2, Canada; alhussan@uvic.ca (A.A.); kbromma@uvic.ca (K.B.); ebozdogan@uvic.ca (E.P.D.B.); WBeckham@bccancer.bc.ca (W.B.); 2 Pancreas Centre BC, Vancouver, BC V5Z 1L8, Canada; ametcalfe@bccrc.ca (A.M.); jkarasinska@bccrc.ca (J.K.); drenouf@bccancer.bc.ca (D.J.R.); 3 BC Cancer, Victoria, BC V8R 6V5, Canada; AAlexander3@bccancer.bc.ca; 4 Department of Pathology & Laboratory Medicine, University of British Columbia, Vancouver, BC V6T 1Z4, Canada; David.Schaeffer@vch.ca; 5 Division of Medical Sciences, University of Victoria, Victoria, BC V8P 5C2, Canada; 6 Centre for Advanced Materials and Related Technologies (CAMTEC), University of Victoria, Victoria, BC V8P 5C2, Canada; 7 Center for Biomedical Research, University of Victoria, Victoria, BC V8P 5C2, Canada; 8 Department of Computer Science, Mathematics, Physics and Statistics, Okanagan Campus, University of British Columbia, Kelowna, BC V1V 1V7, Canada

**Keywords:** pancreatic cancer, gold nanoparticles, uptake, retention, PANC-1, Mia PaCa-2, normal

## Abstract

Pancreatic cancer is one of the deadliest types of cancer, with a five-year survival rate of only 10%. Nanotechnology offers a novel perspective to treat such deadly cancers through their incorporation into radiotherapy and chemotherapy. However, the interaction of nanoparticles (NPs) with cancer cells and with other major cell types within the pancreatic tumor microenvironment (TME) is yet to be understood. Therefore, our goal is to shed light on the dynamics of NPs within a TME of pancreatic origin. In addition to cancer cells, normal fibroblasts (NFs) and cancer-associated fibroblasts (CAFs) were examined in this study due to their important yet opposite roles of suppressing tumor growth and promoting tumor growth, respectively. Gold nanoparticles were used as the model NP system due to their biocompatibility and physical and chemical proprieties, and their dynamics were studied both quantitatively and qualitatively in vitro and in vivo. The in vitro studies revealed that both cancer cells and CAFs take up 50% more NPs compared to NFs. Most importantly, they all managed to retain 70–80% of NPs over a 24-h time period. Uptake and retention of NPs within an in vivo environment was also consistent with in vitro results. This study shows the paradigm-changing potential of NPs to combat the disease.

## 1. Introduction

It is thought that if humans live long enough everyone will eventually develop cancer. The current human lifetime risk of developing cancer is about 40%, and the risk of dying from it is about 21% [1]. According to the World Health Organization, cancer is the second leading cause of death globally, and about one in six deaths is due to cancer. In 2018, the number of cancer cases (excluding non-melanoma skin cancer) exceeded 18 million worldwide, with cancer mortality numbers reached a staggering 9.6 million deaths according to the World Cancer Report. Pancreatic ductal adenocarcinoma cancer (PDAC) is the fourth deadliest cancer by number of deaths worldwide. Its treatment regimens are highly dependent on the cancer stage and location, and include surgery, chemotherapy, and radiation therapy [2,3,4,5]. Despite the advances in radiation therapy, chemotherapy, and surgical procedures in the last decade, pancreatic cancer is still one of the least survivable of all cancer types, with a five-year death rate of 90% [2,3,4,5]. Currently, surgery is the best curative pathway for pancreatic cancer. However, because of the invasive metastatic nature of pancreatic cancer, 80% of patients are not eligible for surgery, and now immunotherapy has emerged as an effective modality to treat such patients [2,6]. On the other hand, one of the major issues in radiotherapy (RT) in treating pancreatic cancer is the close proximity of adjacent organs at risk, resulting in treatment doses being limited by significant tissues’ toxicities, preventing the dose escalation needed for local control [7,8]. With chemotherapy, the presence of the tumor microenvironment (TME), including stroma cells and the extracellular matrix (ECM), prevents propagation of chemotherapeutic drugs denying the delivery of the necessary doses. Hence, the need for a more effective treatment approach is evident. Nanotechnology has demonstrated a remarkable capability to overcome these challenges [8,9,10,11,12]. For instance, nanoparticles (NPs) have shown promising results as radiosensitizer agents in radiotherapy and as vectors for targeted-drug delivery in chemotherapy [6,7,8]. Understanding of the TME and its interactions with NPs is critical in reaping the benefits of nanotechnology, particularly in pancreatic cancer where tumor stroma acts as a barrier.

**Figure 1:**
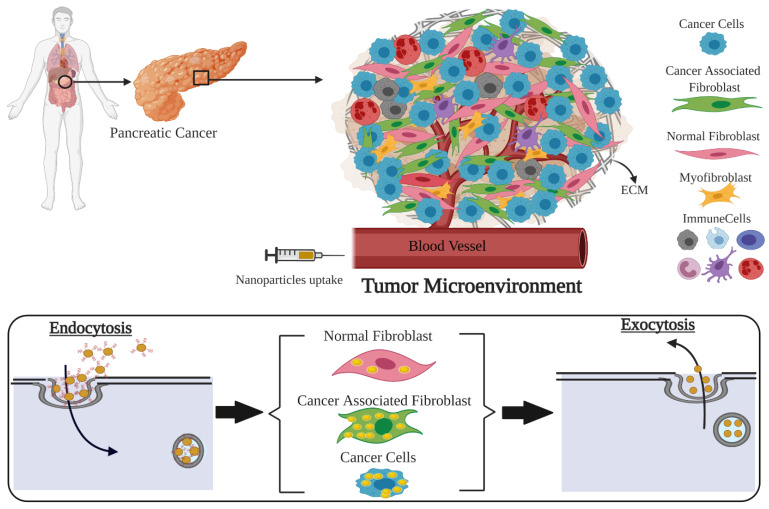
Exploring nano-bio interface in a tumor microenvironment (TME) of pancreatic origin. This schematic demonstrates the complexity of a TME and how nanotechnology can exploit the ability of different cell types to uptake different amounts of nanoparticles (NPs). NPs could be used as radiosensitizers or drug delivery carriers. TME consists of many types of cells. Upon the delivery of NPs into the different type of cells via the bloodstream, cancer cells and cancer associated fibroblasts (CAFs) uptake the highest number of NPs compared to normal cells. The surface-modified NPs enter the cells via endocytosis, and after a certain period of time, leave the cells via exocytosis. The window where cancer cells and CAFs retain the highest number of NPs could be used to deliver specified doses of ionizing radiation to reach a higher therapeutic index.

As illustrated in Figure 1, a successful incorporation of nanotechnology to cancer therapeutics requires a comprehensive understanding of the interface between the TME and NPs. The TME is composed of the ECM along with multiple cell types, including tumor cells, normal fibroblasts (NFs) and cancer-associated fibroblasts (CAFs). It is known that CAFs promote tumor growth while NFs suppress tumor growth [13,14,15,16,17]. NFs are a cell type that produces various essential proteins in the extracellular matrix, are associated with wound healing, and support normal tissue functioning. Unlike NFs, CAFs stimulates tumor growth, support angiogenesis, promote metastasis, play a negative role in tumor surgical excision, and are radio- and chemo-resistant [18,19,20]. Depending on the cancer type and individual human variations, CAFs comprise between 15% and 85% of the total stromal cell population, with roughly 10% of cells being cancer cells [3,21]. In particular, pancreatic tumors form a fibrotic stroma via desmoplasia, characterized by extensive deposition of ECM components and localization and activation of CAFs, which reduces vasculature patency and drug access and alters the anti-tumor immune response [22]. Therefore, CAFs are a predominant component of the stroma and represent an under-explored potential therapeutic target. Despite that, the current interest in use of NPs in cancer therapy is mainly focused on pancreatic tumor cells rather than the TME as a whole. It is yet to be understood how NPs are taken up and retained by other cell types within the TME, such as CAFs and NFs. Retention of NPs is as important as their uptake within individual cell types for their use in drug delivery and radiotherapy [10,11,12]. In this study, we first evaluated the extent of uptake and retention of NPs within these individual cell types from a TME in a monoculture in vitro environment. Our next goal was assessing the extent of accumulation and retention of NPs within a real TME, collectively using an in vivo tumor model and comparing it to our in vitro studies. Therefore, we will address the following fundamental questions in order to exploit the full benefits of cancer nanotechnology to treat pancreatic cancer:

1. Is there a difference between in vitro uptake of NPs in tumor cells, NFs, and CAFs of pancreatic origin?
2. What is the potential of retaining of these NPs within those cells in vitro for efficient delivery of therapeutics?
3. Do we see retention of NPs within tumor tissue in vivo for an extended period of time as being successful for the delivery of therapeutics?

## 2. Materials and Methods

### 2.1. Gold Nanoparticle Synthesis

Spherical gold nanoparticles (GNPs) of 17.73 nm ± 0.17 nm diameter were produced using a citrate reduction method due to its simplicity, its relatively short preparation time, and its low chemical, physical, and environmental dangers. The reducing agent was prepared by adding 0.12 g of sodium citrate tribasic dihydrate (HOC(COONa)(CH_2_COONa)_2_ 2H_2_O) to 10.53 mL of double-distilled water to create a 1% solution. The gold solution was prepared by adding 0.12 g of Tetrachloroauric (III) acid trihydrate (AuCl_4_H·3H_2_O) to 10.35 mL of double-distilled water to create a 1% solution. A total of 300 µL of the 1% gold solution was then added to 30 mL of double-distilled water in an Erlenmeyer flask and was stirred and heated until boiling. Once boiling, 600 µL of the 1% reducing agent was added quickly to the flask and was stirred while boiling for 10 min. The solution’s color gradually changed from clear to a crimson red during this time, indicating the creation of GNPs. The heat was then turned off and the solution was stirred at room temperature for 10 more minutes.

### 2.2. Gold Nanoparticles Functionalization

The negatively charged GNPs surfaces are coated with polyethylene glycol (PEG) at a density of 1 PEG per nm^2^ of GNP surface area. This corresponds to roughly 835 PEG per GNP and the GNP complex is referred as GNP_PEG_. Following PEGylation, a peptide containing integrin binding domain, RGD was added for enhancing the uptake of GNP_PEG_ complex. We added 1 molecule of RGD for every 2 PEG molecules. The final complex is referred to as GNP_PEG-RGD_. The GNP_PEG-CY5-RGD_ used for live cell confocal imaging was PEGylated using 2000 Da PEG and 3400 Da PEG-thiol-CY5 in equivalent amounts, adding up to 835 PEG per GNP and 1 RGD molecule per PEG molecule. PEG of 2000 Da molecular weight was used due to its relatively close molecular weight to RGD peptide (1670 Da).

### 2.3. Gold Nanoparticles Characterization

GNPs, GNP_PEG_, and GNP_PEG−RGD_ shape, size, and concentration were characterized using Perkin Elmer λ 365 ultraviolet visible (UV-VIS) spectrophotometers, while Anton Paar LiteSizer 500 dynamic light scattering (DLS) and ζ potential were used to measure the hydrodynamic radius and surface charge, respectively. UV-VIS shows a measure of the amount of light being absorbed at a particular wavelength by the sample and is dependent on the size and shape of the nanoparticle. Transmission Electron Microscopy (TEM) (Hitachi HF-3300 V, Pleasanton, CA, USA) was used to verify the shape and size of GNPs used in this experiment.

### 2.4. Cell Culture Methodology

Human pancreas cancer cell lines MIA-PaCa-2 (ATCC#: CRL-1420™) and PANC-1 (ATCC^®^ CRL-1469™) were obtained from the American Type Culture Collection. Human cancer-associated fibroblasts (CAF-98) and matched normal pancreas fibroblasts (NPF-98) were derived from resected PDAC tumor tissue from a consenting patient through the Gastrointestinal (GI) Biobank at the Vancouver General Hospital. The study was approved by the University of British Columbia research ethics board (protocol #H20-00948). A small piece of tumor tissue was placed in FGM-2 media (Lonza cc-3132) to support the spontaneous outgrowth of NPs and CAFs. When confluent, the cells were passaged and further propagated in FGM-2 media. During the experiment, all cells were cultured in high glucose Dulbecco’s modified Eagle medium (DMEM; Gibco) enhanced with 10% fetal bovine serum (FBS; Gibco), 1% penicillin/streptomycin (Gibco), and 4 mM of GlutaMax (Gibco). Trypsin–EDTA(Gibco) was used for all cell detachment from flasks and all cell fixations were performed using paraformaldehyde (PFA; Sigma Aldrich, Oakville, ON. Canada). Cell cultures were washed with Phosphate-Buffered Saline (PBS) and all cell line incubations happened at 37 °C with 5% CO_2_.

### 2.5. Image Preparation

Darkfield (DF) coupled with hyperspectral imaging (HSI) CytoViva microscope (CytoViva, Auburn, AL, USA) and Zeiss LSM 980confocal imaging microscopy (Carl Zeiss Microscopy GmbH, Jena, Germany) were used to determine GNP distribution. Fixed cells were imaged for DF and HSI under a 60× objective, while live-cell confocal imaging was done using 60× oil-immersion lens. For DF and HSI, 1 × 10^5^ cells from each cell line were grown on coverslips at the bottom of 6-well dishes with 3 mL of media and were left in the incubator for 24 h. The cell culture media was dosed with 20 µg/mL of GNP_PEG−RGD_ and cells were placed in the incubator for another 24 h. Cells for the uptake study were washed three times with 1 mL of PBS followed by adding 1 mL of 4% paraformaldehyde for fixation and incubated at 37 °C with 5% CO_2_. After a 20 min incubation period, cover slips were rinsed three times with 1 mL of PBS, removed from their wells, and mounted to a glass slide using Permount mounting medium (Fisher Scientific Company, Ottawa, ON. Canada). The media of the cells for the retention study was replaced with fresh media and cells were placed in the incubator for an additional period of 24 h. After the incubation period, cells were washed three times with PBS, fixed, and cover slips were mounted on microscope glass slides for imaging as described previously. For live cell confocal imaging, 1 × 10^5^ cells were grown on 35 mm coverslip-bottom dishes (MatTek, Ashland, MA, USA) with 2 mL of media and were left in the incubator for 24 h. Cells were then dosed with 20 µg/mL of GNP_PEG-CY5-RGD_ (fluorescent CY5 dye; ~651 nm excitation, ~670 nm emission) and were placed in the incubator for another 24 h. Prior to imaging, the cultured media of the cells for the uptake study was substituted with the same amount of colorless media (FluoroBrite DMEM; Gibco, ThermoFisher Scientific, Waltham, MA, USA) and 4 drops of NucBlue^®^ Live reagent (Hoechst^®^ 33342 dye; ~350 nm excitation, ~461 nm emission) (DAPI) in each dish to stain the nucleus of each cell. Cells were incubated for another 20 min before imaging. For the retention study, the media was replaced with fresh media after an incubation time period of 24 h with NPs and cells were placed in the incubator for an additional period of 24 h. Prior to imaging, the cultured media was substituted with colorless media (FluoroBrite DMEM; Gibco) and 4 drops of NucBlue™ Fixed Cell ReadyProbes™ Reagent (DAPI) in each dish to stain the nucleus. Cells in these dishes were then incubated for 20 min before imaging. Acquisition settings were kept the same for both studies.

### 2.6. Quantification of Cellular Uptake and Retention

To measure the gold content in the cell lines, 1 × 10^5^ cells were seeded in 6-well dishes with 3 mL of media for a period of 24 h. All cells were dosed with 20 µg/mL of GNP_PEG−RGD_ and were placed in the incubator for 24 h. For the NP uptake study, cells were washed three times with PBS after 24 h NP incubation time period to remove any GNPs that were not within the cells. In order to remove cells from well for quantification, 1 mL of trypsin was added to each well and placed in an incubator for 5 min. Once cells were detached from tissue culture wells, an additional 5 mL of media was added and mixed well before counting using a hemocytometer. Once the cells were counted, 1 × 10^5^ cells from each well were transferred to glass tubes for quantification.

For the NP retention study, the media containing GNPs was replaced with fresh media following a 24 h NP incubation time period and cells were incubated for an additional period of 24 h to determine their potential of retaining NPs. After the incubation period, cells were also washed 3 times, trypsinized, diluted, and counted for quantification. Similar to the method used in the uptake study, 1 × 10^5^ cells from each well were transferred to glass tubes for quantification.

To process the collected cells for quantification, we added 50 µl of aqua regia (3:1 molar ratio of HCl and HNO_3_(VWR)) to each glass tube and placed them in a mineral oil bath at 90 °C for ~35–45 min until solutions were free of fragments. This was followed by adding 100 μL of hydrogen peroxide (VWR) to each tube before they were returned to the mineral oil bath for ~30 min to confirm full consumption of cell contents. Lastly, the samples were diluted to 2.5% v/v acid content with deionized water, and the amount of gold in each sample was measured by Inductively Coupled Plasma Mass Spectrometry (ICP-MS; Agilent 8800 Triple Quadrupole, Santa Clara, CA, USA) in parts per billion (ppb) or ng/mL. Standard gold chloride solutions were used to produce calibration curves.

### 2.7. Pancreatic Xenograft Model

Human pancreas cancer cell line (MIA-PaCa-2, ATCC#: CRL-1420™) was cultured in Dulbecco DMEM-Dulbecco’s Modified Eagle Medium (ThermoFisher Scientific, Waltham, MA, USA) together with 10% Fetal Bovine Serum (FBS), 100 units/mL penicillin G, and 100 μg/mL streptomycin (Hyclone, Cytiva, Marlborough, MA, USA). Cells were maintained at 37 °C in a humidified atmosphere containing 5% CO_2_. For inoculation in mice, cells were re-suspended in growth medium to the appropriate concentration. For this study, female severe combined immunodeficient (SCID) mice of 6–8 weeks were used. To derive subcutaneous xenografts, 1.5 × 10^6^ tumor cells in a volume of 100 μL were injected using a 28-gauge needle in the lower left dorsal flank. Tumor measurements were converted into tumor volume using (L × W^2^/2), where L and W are the larger and smaller diameters, respectively; tumors were measured every 2 days with calipers. When xenografts reached a volume of ~250 mm^3^, they were randomly divided into groups of 6 for all studies. Each mouse was administered a gold dose of 1 mg/kg in a volume of 100 μL via tail vein using a 28-gauge needle. The concentration of GNPs used for injections was 200 µg/mL and they were suspended in PBS. Animals were monitored for any signs of physical toxicity over the duration of each study. Experiments were conducted in accordance with the Animal Care Committee guidelines of the University Health Network and the approved protocol number is 2979.

### 2.8. Immunohistochemistry

All serial sections (50 µm separation between sections) were cut from frozen block tumor tissue. Sections were stained for CA9 (Rabbit monoclonal; ThermoFisher Scientific, Waltham, MA, USA) and CD31 (provided by Dr. Cameron Koch, University of Pennsylvania, Philadelphia, PA, USA). Secondary antibodies were used alone to control for nonspecific backgrounds. Sections were counterstained with 1 μg/mL DAPI to outline the nuclear area. Images were scanned on the TS4000 (Huron Technologies) at 0.5 μm/pixel. Regions of tumor, necrosis, stroma, and folds were specified, creating a training ruleset for tissue recognition using Tissue Studio (Definiens, Munich, Germany). Cellular analyses included nucleus identification and separation, with objects < 10 µm^2^ being excluded.

### 2.9. Acute and Physical Toxicity Assays

For acute toxicity, blood, liver, kidney, and overall toxicities were measured through proteins, enzymes, metabolites, and plasma electrolytes. Blood samples were collected through terminal cardiac puncture prior to sacrifice. Samples were analyzed using a vetscan2 Autoanalyzer (Applied Biosystems, Foster City, CA, USA). Mice were observed for any physical toxicity over a 50-day period. These physical indicators included body weight changes, dull sunken eyes, rapid/shallow breathing, hunched back, and lethargy.

## 3. Results and Discussion

### 3.1. Characterization of GNP Complexes

**Figure 2:**
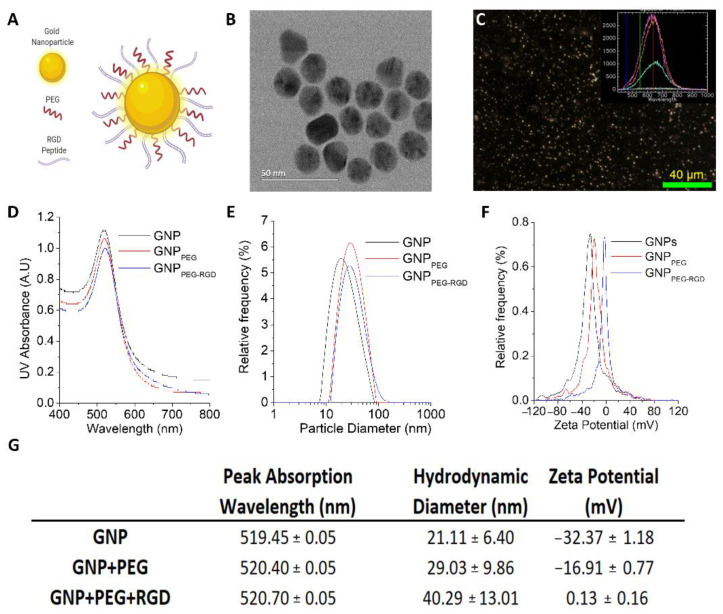
Characterization of GNPs. (**A**) Schematic diagram showing the presence of both PEG and RGD peptide molecules on GNP_PEG-RGD_ complex used for the study. (**B**) TEM images of GNPs with a core size of ~17.73 nm (±0.17 nm). Scale bar is 50 nm. (**C**) DF and HSI images of as-made GNP. Scale bar is 40 µm. For (**D**–**G**), the GNP_PEG_ are GNPs that are functionalized with a polyethylene glycol (PEG) molecule as a stabilizing agent, and the GNP_PEG-RGD_ are GNP that are functionalized with PEG and with RGD peptides to improve GNPs uptake. (**D**–**F**) UV–Visible absorption spectra, hydrodynamic diameter, and ζ-potential measurements of pure GNPs, GNP_PEG_, and GNP_PEG-RGD_, respectively. (**G**) Summary of peak absorption wavelength, hydrodynamic diameter, and mean ζ-potential for pure GNPs, GNP_PEG_, and GNP_PEG-RGD_. (Note: the error is represented by the standard deviation over three different measurements).

GNPs were used as our model NP system since they have been successfully tested as radiosensitizers and drug carriers in radiotherapy and chemotherapy, respectively [23]. The optimization of GNP physical properties (size, shape, surface properties, etc.) is an important factor in the proper utilization of nanotechnology in combined cancer therapy to maximize the beneficial effects. Unfunctionalized GNPs naturally bind to plasma proteins at their surface, enabling their cellular uptake into the cell [24]. However, this makes them vulnerable to attacks from the immune system which clears them from the system, thus causing GNPs to have a short bloodstream circulation lifetime [25]. To overcome this problem, GNP surfaces are coated with polyethylene glycol (PEG) to reduce their binding to the antibody proteins that target foreign objects for clearance. The functionalization process of GNPs with PEG, i.e., PEGylating, results in a complex referred to as GNP_PEG_ [26,27]. This process allows GNP_PEG_ to avoid the immune system as a nonforeign object and extend its blood circulation [28]. The downfall of PEGylating is the reduction in endocytosis of GNPs into cancer cells [29]. Therefore, GNPs were also coated with peptides containing an integrin binding motif known as Arginine-Glycine-Aspartic Acid (RGD)-motif to create GNP_PEG-RGD_ (Figure 2A). The advantage of using peptides to other molecules such as antibodies is their smaller size, thus giving the opportunity for other molecules to be attached to the system as needed for improved future approaches [30,31]. Conjugation of PEG on NP surface facilitated not only the stability for conjugation of RGD peptides to prevent aggregation, but also enhanced the biocompatibility needed for in vivo studies. We used a capping density of ~1 PEG/nm^2^ surface area, which is consistent with other studies [26].

Among the size range from 10 to 100 nm, GNPs of diameter 50 nm seem to have the highest cellular uptake in vitro [32]. However, when they are functionalized with both PEG and RGD peptides of similar size, smaller NPs have the highest cellular uptake [29]. The interaction of RGD peptide with cell surface receptors is enriched for smaller NPs due to their higher surface curvature. Furthermore, these small size NPs have shown better penetration in three-dimensional tissue structures which will have a significant effect in their distribution within tumor tissue [33]. Therefore, we opted for GNPs of 15 nm diameter for this study. Transmission electron microscopy (TEM) images of the as-made GNPs are displayed in Figure 2B and Figure S1. The average core diameter of the NPs was measured to be 17.73 nm ± 0.17 nm. Higher reflectivity of visible light by GNPs allowed us to visualize them using darkfield and hyperspectral imaging, as shown in Figure 2C. The inset Figure 2C shows the spectrum collected from GNPs. The same technique was used to further verify GNP uptake within cells, as discussed later.

In order to determine the size, shape, and concentration of the GNPs and GNP complexes, we also used UV-VIS spectroscopy (Figure 2D), dynamic light scattering (DLS) (Figure 2E), and ζ-potential measurements (Figure 2F). The data are also summarized in the table in Figure 2G. The size and concentration of the GNPs was estimated using UV-Vis spectrometry [26]. According to previous studies, UV-Vis is found to be an accurate measurement of the concentration [34]. The ratio of the peak absorbance of the surface plasmon resonance spectrum to the 450 nm absorbance led to an approximate diameter of 14–16 nm for GNPs. With conjugation of molecules onto the GNP surface, a slight red shift in the peak absorbance was seen, but the general shape of the spectrum did not change appreciably. This is due to the fact that the added PEG and RGD peptide molecules on the GNP surface are relatively smaller in size (PEG: 2 k and RGD peptide: 1.7 k) compared to GNPs. However, there was a significant different in surface properties of GNP with the addition of the PEG and RGD peptides, as seen by DLS and ζ-potential measurements. DLS confirmed the hydrodynamic diameter of as-made GNPs to be 21.11 nm with a polydispersity index of 6%, while the ${\mathrm{GNP}}_{\mathrm{PEG}-\mathrm{RGD}}$ complex had a diameter of 40.29 nm and a polydispersity index of 13%. This increase in the hydrodynamic diameter is consistent with conjugation of the different molecules on the surface. The ζ-potential of the as-made GNPs and ${\mathrm{GNP}}_{\mathrm{PEG}-\mathrm{RGD}}$ complex was measured to be −32.37 mV and 0.13 mV, respectively. Initial negative charge of GNPs is due to the negatively charged citrate molecules present as a cap on the surface. During the functionalization process, citrate molecules on the surface are replaced with neutral PEG molecules and the positively charged RGD peptides resulting in a significant change in the ζ-potential. The ${\mathrm{GNP}}_{\mathrm{PEG}-\mathrm{RGD}}$ complex was also measured for stability in tissue culture media for a period of 24 h to mimic the in vitro environment. The hydrodynamic diameter and ζ-potential of the ${\mathrm{GNP}}_{\mathrm{PEG}-\mathrm{RGD}}$ complex did not vary significantly after the incubation period, as illustrated in Figure S1. Previous studies have also shown that GNPs tagged with ~1 PEG/nm^2^ surface area demonstrated the best stability, which is the capping density employed in this study [26].

### 3.2. Cellular Uptake of GNP Complexes in Tumor Cells, Normal Fibroblasts (NFs), and Cancer-Associated Fibroblasts (CAFs) in Monolayer Cell Cultures

**Figure 3:**
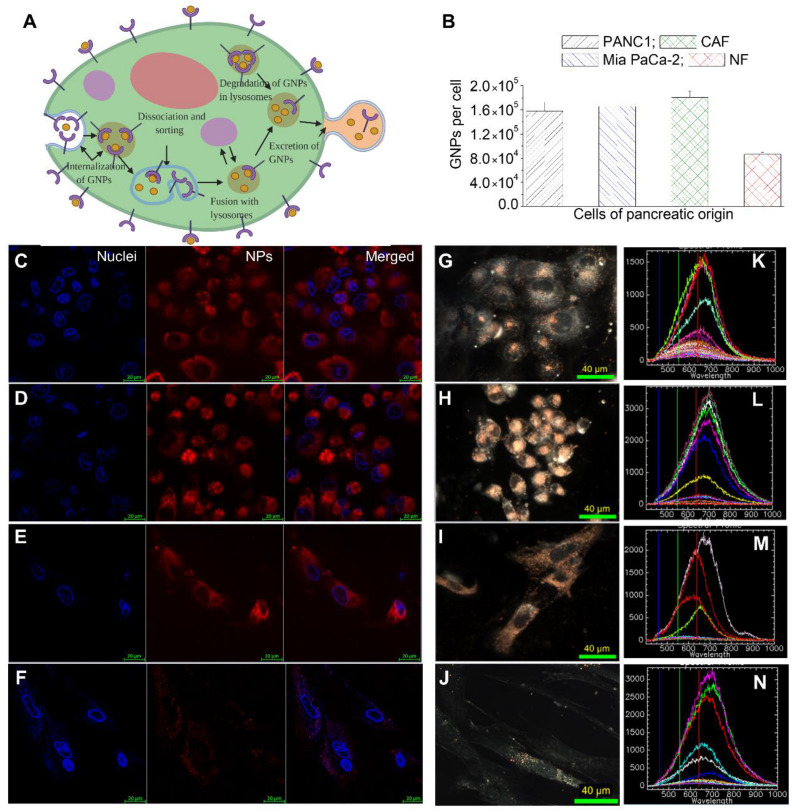
Gold Nanoparticles (GNPs) uptake in cancer cells vs. normal cells. (**A**) A schematic diagram explains the path of GNPs within a cell. (**B**) Quantification of GNPs per cell in PANC-1, Mia PaCa-2, CAFs, and NFs. (**C**–**J**) Visualization of intracellular NP distribution in PANC-1 (first row), Mia PaCa-2 (second row), CAFs (third row) and NFs (fourth row) using confocal imaging (**C**–**F**) left-most panel (nucleus); middle panel (NPs); right-most panel (merged image), and dark-field (**D**,**F**) microcopy (**G**–**J**). (**K**–**N**) Hyperspectral Imaging (HSI) spectra collected from GNP clusters (bright specs) in images from G to J. Scale bars are 20 µm and 40 µm for confocal and DF, respectively.

GNPs enter cells mostly via receptor-mediated endocytosis (RME). The RME process of NPs occurs through interactions between the ligands on the surface of the NP and receptors on the cell membrane. In this study, we used the RGD peptide as a targeting ligand [35]. This was used to exploit the fact that many cancer cells overexpress integrins on their surface, thus binding GNPs with RGD facilitates their receptor-mediated endocytosis specifically in cancer cells. Cell surface receptors bind to ligand molecules on the surface of NPs, such as RGD, causing membrane wrapping of the NP with a corresponding increase in elastic energy [32,36]. The receptor-ligand interaction immobilizes receptors, causing configurational entropy to be reduced. More receptors diffuse to the wrapping site driven by the local reduction in free energy, allowing the membrane to wrap completely around the particle. Receptor-mediated endocytosis is an energy-dependent process. The path of the NPs within the cell is explained in Figure 3A. Once GNPs are bound to the receptors on the surface of the cell, membrane invagination occurs followed by trapping of GNPs in endosomal vesicles. The internalized GNPs are sorted inside the vesicle and eventually fuse with lysosomes. GNPs are then excreted out of the cell. This is called the endo-lyso pathway.

Most NP uptake studies have mainly focused on tumor cells. However, TME consists of many other cell types which could significantly influence the outcome of NP-based therapeutics. Among the cells present in TME, our focus was on NFs and CAFs in addition to tumor cells. This is mainly due to the anti-tumorigenic properties of NFs and the tumorigenic properties of CAFs. CAFs promote the proliferation of a tumor through release of cytokines and chemokines, while NFs suppress it [18,19,20,37]. The cell lines used in this study are PANC-1 (a human pancreatic cancer cell line derived from a pancreatic carcinoma of ductal origin), MiaPaca2 (a human pancreatic cancer cell line derived from a pancreatic carcinoma), CAF-98 (a patient-derived pancreatic cancer-associated fibroblast) and NPF-98 (a normal pancreatic fibroblast).

In order to map the GNP uptake cross section among these four cell lines, we incubated cells with ${\mathrm{GNP}}_{\mathrm{PEG}-\mathrm{RGD}}$ complex at a concentration of 20 µg/mL (or ~0.8 nM) for 24 h. A GNP concentration of 20 µg/mL is shown to be well within the tolerable doses for in vivo administration, with a goal in mind for future clinical applications [38,39,40,41]. The uptake of the ${\mathrm{GNP}}_{\mathrm{PEG}-\mathrm{RGD}}$ complex per cell was quantified using inductively coupled plasmon mass spectroscopy (ICP-MS) (Figure 3B). GNP uptake in tumor cells and CAFs were comparable while GNP uptake in NFs was two-fold less compared to that of tumor cells and CAFs. The lower uptake of GNPs in NFs and higher uptake in tumor cells and CAF-98 is consistent with previous studies where CAFs and NFs of melanoma origin were compared to tumor cells of breast and cervical origins [42,43]. Only cells of pancreatic origin were used in this study. Furthermore, to have a more meaningful comparison, the pancreatic CAFs and NFs used were obtained from the same patient. One of the reasons for the difference in NP uptake between tumor cells, CAFs, and NFs could be due to the receptor expression of integrin-binding domain RGD among these cells. We used GNPs labeled with RGD peptide, and previous studies show that this particular peptide can improve endocytosis and can effectively target tumors [27,30]. However, the disparity in integrin expression between these cells is not fully elucidated yet. It was noticeable that the extent of NP uptake in CAFs was comparable to that of tumor cells but much lower compared to NFs, even though CAFs are typically generated from NFs [44]. This is very promising in terms of targeting NPs for cancer therapeutics. CAFs promote virtually every aspect of the hallmarks of cancer. For example, the presence of CAFs in cancer specimens from patients has been associated with a poor prognosis in multiple cancers [45,46,47].

Based on our quantification data, we believe that we can deliver therapeutics that target tumor cells and CAFs very effectively, not only to kill tumor cells but also to silence CAFs which promote tumor growth. This type of novel therapeutic is very much needed in treating pancreatic cancer where survival is very low. A recent study has demonstrated the feasibility of silencing CAFs of melanoma origin using both GNPs and radiation [43]. GNPs used as a radiosensitizing agent in radiotherapy and the ability of CAFs to take in GNPs similar to tumor cells result in their vulnerability during radiotherapy. These novel approaches will shed light on treating pancreatic cancer, which has an extensive deposition of ECM combined with a high activation of CAFs [22]. Imaging techniques were also utilized to further verify our quantitative data, as shown in Figure 3B, and to map the distribution of GNPs within cells, as illustrated in Figure 3C–J. Both confocal (leftmost panel (Figure 3C–E); nucleus is stained blue and NPs are in red) and DF images (Figure 3F–J) further solidify our quantification data in Figure 3B. Tumor cells (PANC-1 (panel C) and Mia PaCa-2 (panel D)) and CAFs (panel E) had a significantly higher NP presence compared to NFs (Panel F). We were also able to confirm the presence of GNPs using the spectral mapping feature of HSI, as illustrated in the rightmost column of Figure 3K–N. Reflectance spectra of GNPs was obtained from a few selected bright spectra and we confirmed that they were from GNP clusters using the data available in the imaging library. This technique is very useful since it is not necessary to optically label GNPs in contrast to confocal imaging.

### 3.3. Retention of GNP Complexes in Tumor Cells, Normal Fibroblasts (NFs), and Cancer-Associated Fibroblasts (CAFs) in Monolayer Cell Cultures

GNPs are taken up by cells via the endocytosis process where they are trapped in endosomal compartments upon entering, get processed through fusing with lysosomes, and are excreted to the extracellular medium, as explained in Figure 3A. After few hours of cells being in contact with GNP-containing media, an equilibrium between endocytosis and exocytosis processes is reached, resulting in a plateau in cellular uptake where the presence of NPs within cells is measured over an extended period of time [48]. The question then arises regarding what happens if the NPs are removed from the medium. The same four cell lines that were used in the uptake experiments were used to monitor the ability of NPs retention over a period of 24 h. The GNP-containing media was substituted with fresh media and all cells were incubated for an additional period of 24 h. Thus, the total amount of time between the introduction of GNPs into the cells to quantification was 48 h.

**Figure 4:**
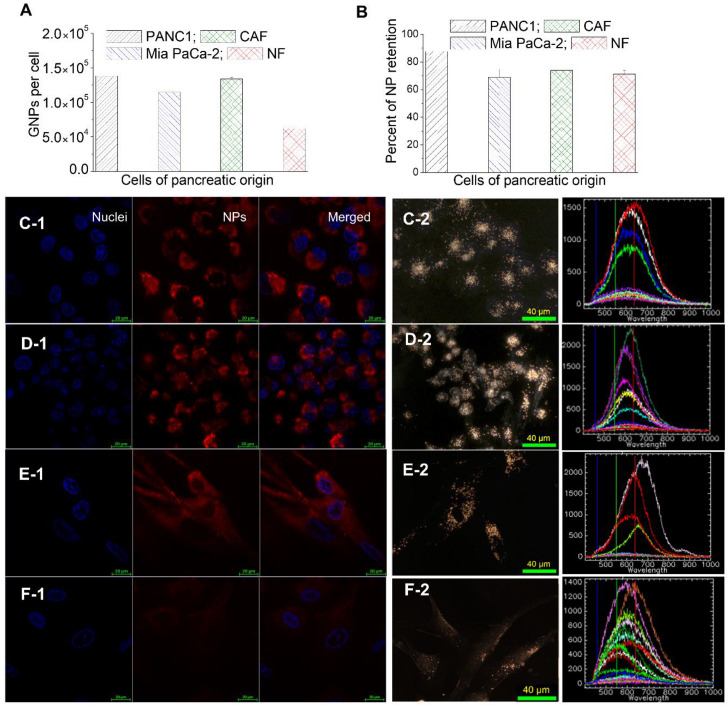
Retention of GNPs in cancer cells vs normal cells. (**A**) GNPs retained per cell in PANC-1, Mia PaCa-2, CAFs, and NFs. (**B**) Percent of NP retention in PANC-1, Mia PaCa-2, CAFs, and NFs. (**C**–**F**) Confocal (leftmost panel; nucleus is stained in blue and NPs are in red), DF images (middle panel; bright specs represent GNP clusters), and HSI spectra (rightmost panel; spectra collected from bright specs as compared to background) of GNPs internalized in PANC-1 (**C**), Mia PaCa-2 (**D**), CAFs (**E**), and NFs (**F**). Scale bars are 20 μm and 40 μm for confocal and DF, respectively.

A clear reduction in the number of GNPs was apparent in all four cancer cell lines compared to normal media. This could be attributed to two reasons, the first being the redistribution of GNPs from the parent cell to the daughter cells following cell division, and the second reason being the nonequilibrium excretion of GNPs from cells via exocytosis without having enough GNPs getting into the cell due to lack of GNPs in the surrounding media [42]. Even though the number of GNPs in cells 24 h post-incubation with fresh media decreased, tumor cells and CAFs were still able to have more NPs in them compared to NFs (Figure 4A). The retention percentage of GNPs in tumor cells, CAFs, and NFs was approximately the same at 70–80% (Figure 4B). The trend in intracellular retention of NPs is consistent with previous studies where a similar GNP complex was used with different cell lines [42,43]. Typically, GNPs get into the cell via RME where they are caught in endosomes before being fused and localized in lysosomes for handling, and finally exiting the cell through exocytosis where they are excreted via fusion with the cell membrane, as illustrated in Figure 3A. In this process, the majority of GNP are concentrated in endosomes and lysosomes rather than the cytoplasm or the nucleus [49]. Based on previous studies, these NPs get excreted at a much faster rate [48]. However, conjugating GNPs’ surfaces with RGD peptides promotes GNPs to discharge into the cytoplasm from endosomes following endocytosis [50,51]. This gives GNPs a longer time to spend in cells, which in return decreases the rate of exocytosis, thus increasing their percent of retention (Figure 3A). The particular RGD peptide that we used in our study was also used for releasing NPs from endosomes and lysosomes to facilitate nuclear targeting [35,50]. This extended intracellular retention of GNPs in cancer cell lines could be exploited in combined radio- and chemo-cancer therapies. Despite the importance of understanding the retention beyond the 24 h period used in this study, studying such long-term effects is not feasible due the significant difference in doubling time between these cells and the dilution of NPs between the dividing cells [52]. The combination of these two effects may not provide us with a reliable conclusion regarding NP retention over a longer period of time.

### 3.4. The Dynamics of GNP Distribution and Retention within Tumor Tissues In Vivo

Our in vitro studies discussed earlier (Figure 3 and Figure 4) demonstrated the ability of cells to retain GNPs at a significantly high percentage (70% to 80%) even after 24 h of the source of NP (i.e., the NPs-containing media) was removed. This result is very significant only if it can be reproduced in a real tumor environment since it is generally accepted that in vitro results cannot be extrapolated directly to an in vivo environment. To compare our in vitro results to an in vivo setting, we used one of our pancreatic tumor cell lines, Mia PaCa-2, to generate an in vivo tumor model. Unlike in vitro where NPs are presented directly to the cells via their media, NPs face many barriers in vivo before reaching the cells.

**Figure 5:**
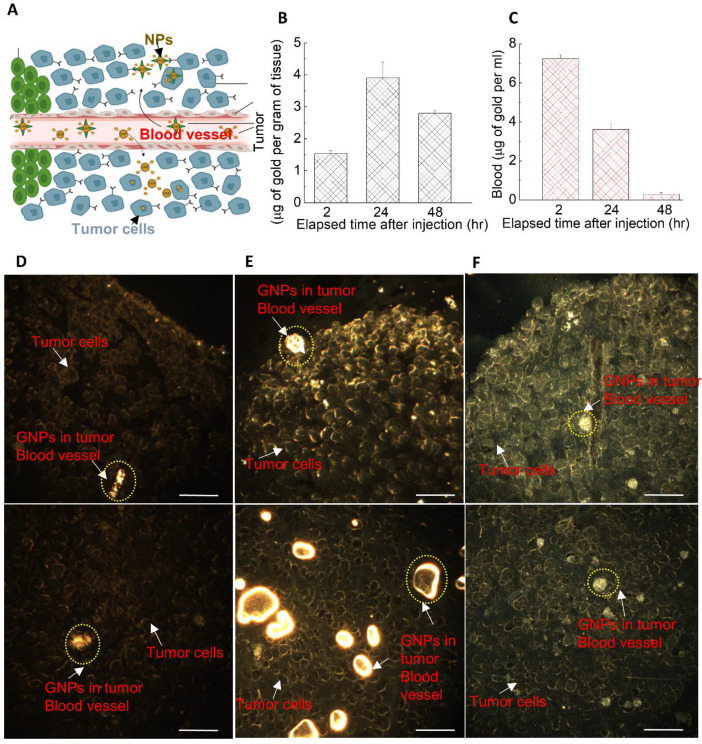
The dynamics of GNP distribution and retention in tumor. (**A**) A schematic diagram showing the escape of NPs from leaky blood vessels to tumor tissue. (**B**–**C**) Quantification of GNPs in tumor and blood (in circulation) over a period of 24 h. (**D**–**F**) GNPs distribution in tumor tissue after 2, 24, and 48 h of injection, respectively. Top and bottom rows represent tumor periphery and interior, respectively. Scale bar is 40 µm.

As illustrated in schematic Figure 5A, tumors’ leaky vasculatures and ineffective lymph systems facilitate the systemic circulation of NPs within the tumor, resulting in extravagation and accumulation of NPs in tumor cells [53,54]. This process is also known as the enhanced permeability and retention (EPR) effect. Our pharmacokinetic studies of GNPs in a pancreatic cancer model showed that the accumulation of the GNPs in the tumor was highest at 24 h post injection (see Figure 5B), while it was highest in blood (in circulation) at a 2 h time point. It was also evident that NPs clearance from the tumor (Figure 5B) was lower than NPs clearance from the blood in circulation (see Figure 5C). This indicates that GNP retention in the tumor is longer than GNP clearance from the circulatory system. Even after 48 h of post-NP injection, tumor tissue retained ~75% of the optimum accumulation reached after 24 h. This is consistent with the outcomes of our in vitro retention studies. We were able to verify our quantification data (in Figure 5B) with qualitative data as illustrated in Figure 5D–F. Similar to our in vitro studies, we were able to visualize the presence of GNPs in tumor tissue, which included tumor cells and tumor blood vessels using DF imaging. The bright yellow structures represent GNP clusters in tumor tissue cross-sections, as pointed out by arrows in those images. We were able to visualize both tumor cells and cross-sections of tumor blood vessels (see image panels of Figure 5D–F). After 2 h of NP injection, we did not see a significant presence of NPs in tumor tissue (Figure 5D). There was a significant increase in NPs presented in the tumor at a 24-h time point (Figure 5E). Even after 48 h of NP injection, we could still see NPs remaining within the tumor tissue (Figure 5F). However, the intensity of GNPs within tumor blood vessels reduced dramatically as NPs were no longer present at a significantly high percent within the circulation system, as explained in Figure 5C. Therefore, our quantification data presented in Figure 5B,C was further solidified with qualitative image panels in Figure 5D–F and Figure S2. It also important to differentiate that Figure 5C represents the NPs within the circulating blood. The presence of NPs in tumor blood vessels could be assessed only qualitatively using the images we had. The data presented in Figure 5B shows the NPs in both tumor tissue and tumor blood vessels collectively. Additional images in Figures S3 and S4 show the presence of GNPs in tumor blood vessels 24-h and 72-h post injection.

Modification of size and surface properties of NPs allowed us to have a significant number of GNPs present even 48-h post injection, which would facilitate better delivery of nanomedicines in future cancer treatments. In one of the pioneering studies using GNPs as radiosensitizers, radiation treatment had to be performed minutes after the injection of NPs since NPs were excreted much faster [55]. Our approach of modifying the surface properties of GNPs would enable higher circulation time, as well as active targeting of tumor cells once they reach the tumor tissue. This hypothesis has been further supported by another in vivo study where GNPs decorated with an RGD-like peptide had a four-fold higher accumulation within the tumor compared to that of uncoated particles [56]. The safety and efficacy of a multiple dosing approach has been demonstrated using GNPs and has been found to increase particle accumulation in tumors [57]. Multiple dosing could potentially enhance the efficacy of radiation dose enhancement in radiotherapy and drug delivery in chemotherapy.

To generate our in vivo tumor model for this study, we used Mia PaCa-2, which is a standard tumor cell line. However, this type of tumor model may lack the complex nature of TME where CAFs play a significant role. Therefore, our future studies will include generating tumors from patient-derived xenograft models of pancreatic origin or co-injection of both tumor cells and CAFs to create real tumor-like environments for testing nanomedicines in a more meaningful manner. This type of model will allow us a better comparison between our in vitro and in vivo studies for advancing cancer medicine for future clinics.

## 4. Conclusions

Despite the latest advancements in radiotherapy, chemotherapy, immunotherapy, and surgical techniques for the management of pancreatic cancer, the majority of pancreatic cancer patients will die from the disease. Therefore, new treatment strategies are undoubtedly needed. One approach is to integrate cancer nanotechnology into current curative modalities, such as in radiotherapy to enhance the local radiation dose, and in chemotherapy to facilitate drug delivery. Most studies have so far focused on tumor cells. However, cancer-associated fibroblasts (CAFs) are key players in the tumor microenvironment (TME). They are responsible for promoting growth and metastasis of the tumor. As such, CAFs are associated with poor prognosis and have emerged as a focus of anticancer research. Our in vitro study shows the potential of targeting GNPs not only to tumor cells but also to CAFs. Furthermore, NP uptake by CAFs was over 10% higher than that of tumor cells. This will play a significant role in reducing the activity of CAFs when GNPs are used as radiosensitizing agents or as drug carriers. Most importantly, the percent retention of NPs 24-h post injection in CAFs and tumor cells was over 70%. The ability of CAFs and tumor cells to retain most NPs over a 24-h period allows for a better flexibility in the delivery of NP-based therapeutics. It was also noticeable that uptake of NPs by NFs was two-fold less compared to CAFs. This could shed light on concerns regarding normal tissue toxicity due to NPs. Results of our in vivo study were also very promising as we noticed 10% of the injected NP dose within the tumor 24-h post injection. After 48 h, over 70% of the NPs still remained within the tumor. We noticed a similar behavior of GNPs retention in our in vitro study as well. Our study concludes that NP-based targeting of both tumor cells and CAFs could be used as a smart novel strategy towards improving current anti-cancer therapies which include radiotherapy and chemotherapy. For an even better representation of a patient TME, our future in vivo studies will involve generating tumors from patient-derived xenograft models. We believe that a collective effort among biomedical researchers, radiation oncologists, medical oncologists, and pathologists could bring these novel therapeutics to the forefront for developing an effective strategy to increase the cure-rate in patients with pancreatic cancer.

## Data Availability

The data presented in this study are available in this article and supplementary material.

## Supplementary Materials

The following are available online at https://www.mdpi.com/article/10.3390/curroncol28030183/s1, Figure S1. Characterization of gold nanoparticles, Figure S2. Uptake and retention of GNPs in tumor tissue, Figure S3. GNP distribution in blood vessels and cells within the tissue after 24 h of GNP injection, Figure S4. GNP distribution in blood vessels and cells within the tissue after 72 h of GNP injection.
