# Supplementary material for: Investigation of Nano-Bio Interactions within a Pancreatic Tumor Microenvironment for the Advancement of Nanomedicine in Cancer Treatment
Source: Curr Oncol. 2021 May 24;28(3):1962–79. doi: 10.3390/curroncol28030183 (PMC8161808; doi:10.3390/curroncol28030183)
Supplement: Supplementary file 1 [file curroncol-28-00183-s001.zip › curroncol-1193855-supplementary.pdf]

# Supplementary Materials

# Investigation of Nano-Bio Interactions within a Pancreatic Tumor Microenvironment for the Advancement of Nanomedicine in Cancer Treatment

**Abdulaziz Alhussan [1], Kyle Bromma [1], Ece Pinar Demirci Bozdoğan [1], Andrew Metcalfe [2], Joanna Karasinska [2], Wayne Beckham [1,3], Abraham Alexander [3], Daniel J. Renouf [2], David F. Schaeffer [4] and Devika B.**

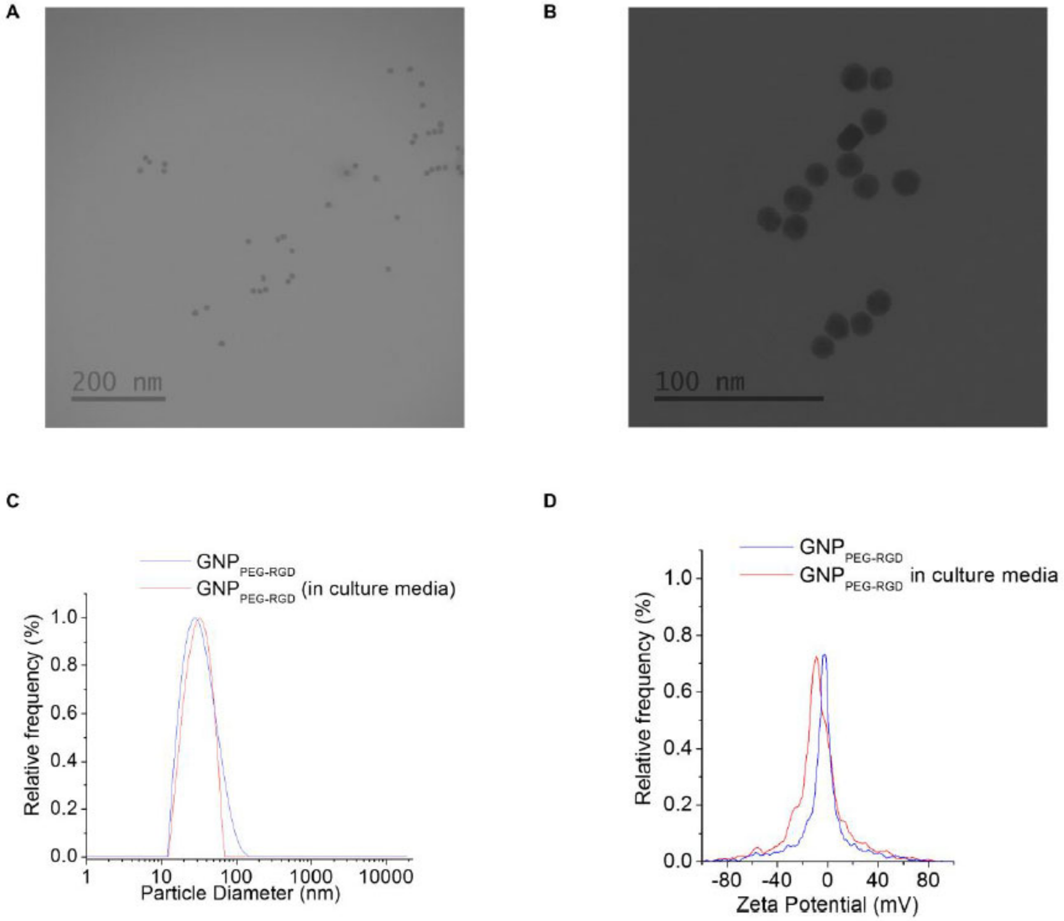


**Figure S1.** Characterization of gold nanoparticles. A–B) Transmission electron microscopy images of as-made gold nano-parti-cles. C–D) Verifying the stability of GNPs in tissue culture media for an incubation time period of 24 hrs using dy-namic light scattering and zeta-potential measurements, respectively.

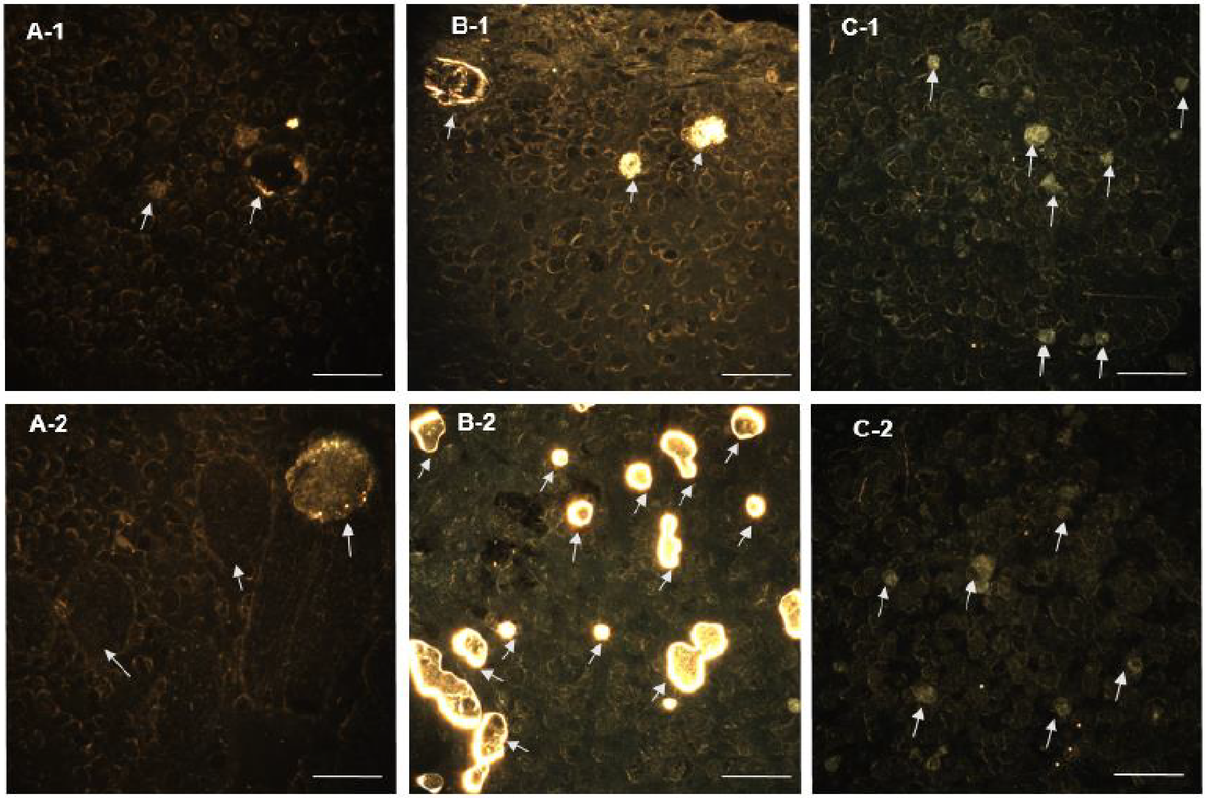


**Figure S2.** Uptake and retention of GNPs in tumor tissue. **A–C)** Distribution of GNPs in a tumor after 2, 24, and 48 hrs after intravenous injection of GNPs, respectively. Scale bar is 40 μm. Cross-section of blood vessels within the tissue are marked with arrows.

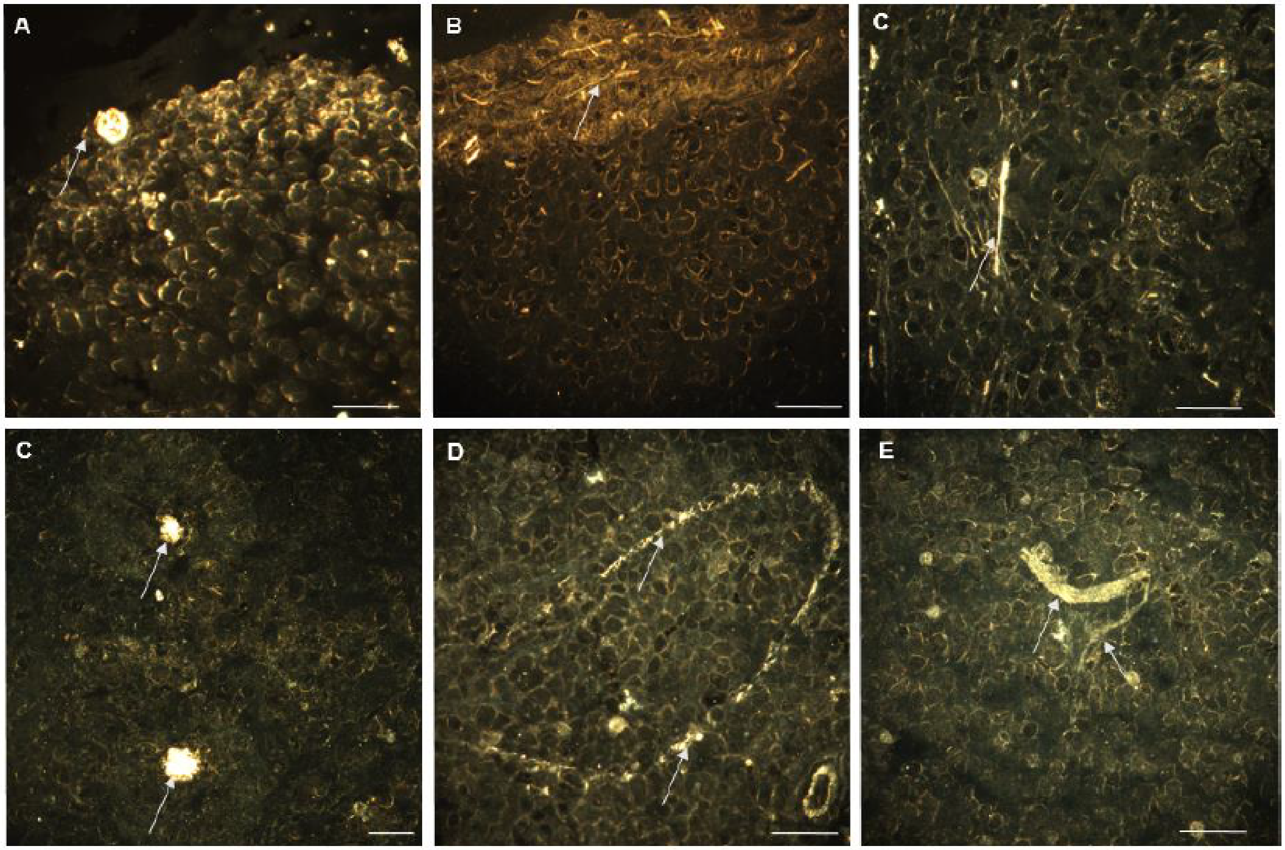


**Figure S3.** GNP distribution in blood vessels and cells within the tissue after 24 hrs of GNP injection. Scale bar is 40 μm. Cross-section of blood vessels within the tissue are marked with arrows. .

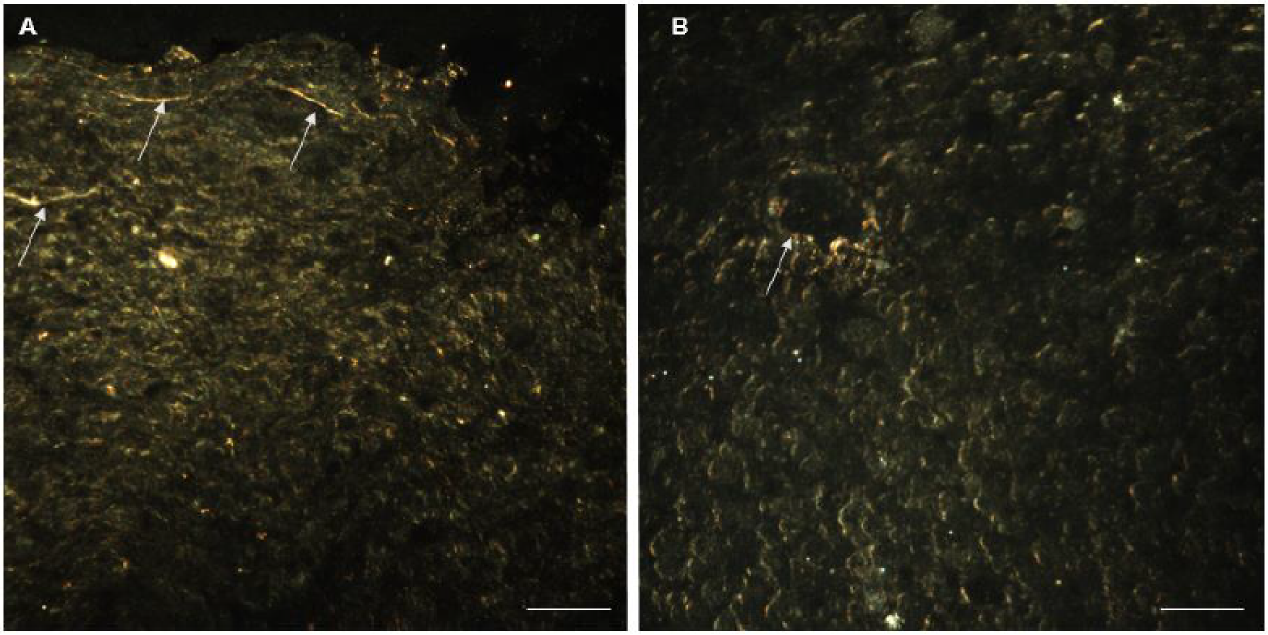


**Figure S4.** GNP distribution in blood vessels and cells within the tissue after 72 hrs of GNP injection. Scale bar is 40 μm. Cross-section of blood vessels within the tissue are marked with arrows.
